# Guidelines for pre-analytical conditions for assessing the methylation of circulating cell-free DNA

**DOI:** 10.1186/s13148-021-01182-7

**Published:** 2021-10-18

**Authors:** Mohammad Amin Kerachian, Marjan Azghandi, Sina Mozaffari-Jovin, Alain R. Thierry

**Affiliations:** 1 Medical Genetics Research Center, Mashhad University of Medical Sciences, Mashhad, Iran; 2 Department of Medical Genetics, Faculty of Medicine, Mashhad University of Medical Sciences, Mashhad, Iran; 3 Cancer Genetics Research Unit, Reza Radiotherapy and Oncology Center, Mashhad, Iran; 4 Department of Animal Science, Faculty of Agriculture, Ferdowsi University of Mashhad, Mashhad, Iran; 5 IRCM, Institute of Research in Oncology of Montpellier, Montpellier, France; 6 INSERM, U1194, Montpellier, France; 7 University of Montpellier, Montpellier, France; 8 ICM, Regional Institute of Cancer of Montpellier, Montpellier, France

**Keywords:** Epigenetics, Liquid biopsy, Pre-analytical, Circulating tumor DNA, Biomarkers

## Abstract

Methylation analysis of circulating cell-free DNA (cirDNA), as a liquid biopsy, has a significant potential to advance the detection, prognosis, and treatment of cancer, as well as many genetic disorders. The role of epigenetics in disease development has been reported in several hereditary disorders, and epigenetic modifications are regarded as one of the earliest and most significant genomic aberrations that arise during carcinogenesis. Liquid biopsy can be employed for the detection of these epigenetic biomarkers. It consists of isolation (pre-analytical) and detection (analytical) phases. The choice of pre-analytical variables comprising cirDNA extraction and bisulfite conversion methods can affect the identification of cirDNA methylation. Indeed, different techniques give a different return of cirDNA, which confirms the importance of pre-analytical procedures in clinical diagnostics. Although novel techniques have been developed for the simplification of methylation analysis, the process remains complex, as the steps of DNA extraction, bisulfite treatment, and methylation detection are each carried out separately. Recent studies have noted the absence of any standard method for the pre-analytical processing of methylated cirDNA. We have therefore conducted a comprehensive and systematic review of the important pre-analytical and analytical variables and the patient-related factors which form the basis of our guidelines for analyzing methylated cirDNA in liquid biopsy.

## Introduction

Circulating cell-free DNA (cirDNA) is now a significant blood or lymph biomarker in non-invasive liquid biopsy [1]. Liquid biopsy has recently shown considerable promise as a diagnostic technique with many benefits over traditional invasive techniques [2]. It offers new prospects for the diagnosis and treatment of diseases such as clinical infectious diseases [3], genetic disorders [2, 4], hematological diseases [5], human microbiome identification [6], early detection of organ rejection [7], and mostly cancer [8, 9]. It may even have applications in non-pathogenic areas, such as in the evaluation of the physical exercise [10].

At present, tissue biopsies remain the gold standard for cancer detection and molecular characterization. However, these traditional (solid biopsy) sampling approaches have some drawbacks, including difficulties in collecting sufficient amounts of biomaterial, sampling prejudice due to tumor genetic heterogeneity, and procedural complications, [11] as seen for some solid tumors such as lung and brain cancer [12]. To tackle such limitations, liquid biopsies have emerged as a vital alternative to tissue biopsies [13]. Recent experiments have demonstrated the considerable potential of liquid biopsies to uncover new biomarkers [14–16]. Given the significant clinical implications of this, liquid biopsies have received much attention in recent years [11, 17–19]. The role of genetic alterations in cancer, and the identification of driver mutations in proto-oncogenes and tumor suppressor genes identified in circulating tumor DNA (ctDNA), have been described in a large number of studies [20–22]. Despite the fact that ctDNA was discovered many years ago, the absence of an appropriate pre-analytical technique means that few cirDNA-based mutation detection assays have so far made their way into clinical diagnosis [23] and recent studies have highlighted the absence of any standard method for the pre-analytical analysis of cirDNA, in particular methylated cirDNA. Indeed, only Meddeb et al. have performed a systematic review providing guidelines for nuclear cell-free DNA (cfDNA) extraction suitable for mutation detection [24].

**Fig. 1:**
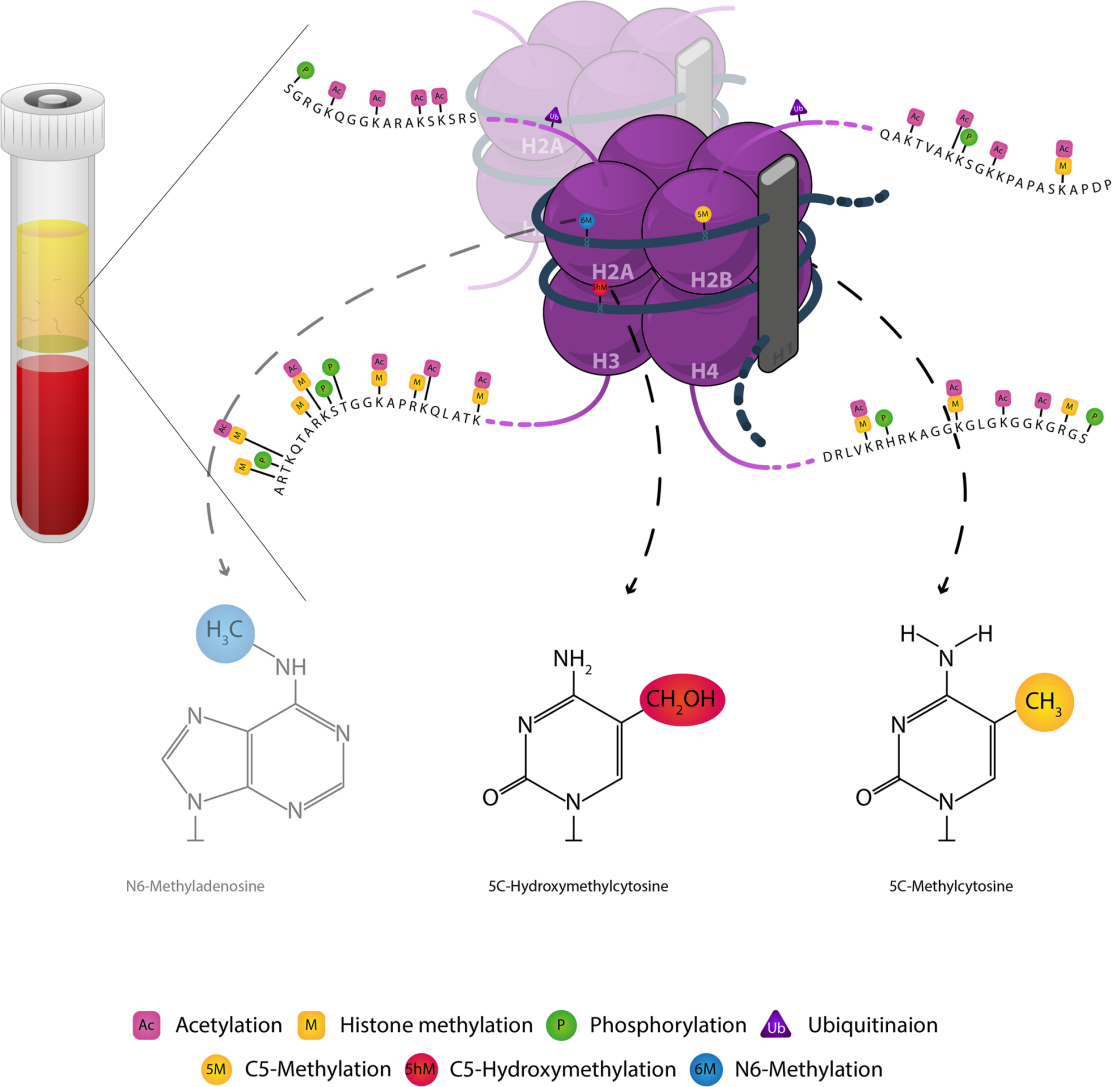
Schematic epigenetic modifications in cirDNA that regulate chromatin organization and gene expression. Epigenetic processes mainly consist of histone modifications including acetylation, phosphorylation, ubiquitination and histone methylation. Besides, DNA methylation consists of C5-methylation, C5-hydromethylation and N6-methylation. Black bold and purple thin strands display double strand DNA and histone tails, respectively

In addition to driver mutations, epigenetic modifications particularly DNA methylation are among the most extensive genomic aberrations which occur during carcinogenesis [25, 26] as well as in genetic diseases [27]. These modifications are correlated with biological processes via the modulation of gene expression [28]. Epigenetic processes mainly consist of histone modifications and DNA methylation [29, 30] (Fig. 1). The altered forms of DNA methylation are early events in many diseases [31–33] such as breast [34] and colorectal cancers (CRC) [9, 35], and as such are important for early cancer detection and cancer screening [36]. Hence, the analysis of cirDNA methylation is considered a reliable and versatile method for the diagnosis and prognosis of cancer [37–39].

Methylation alterations can be categorized as focal hypermethylation and global hypomethylation in cancer [40–42], as well as the hyper/hypomethylation of most gene promoters in other diseases such as metabolic diseases [43, 44], Alzheimer’s disease [45–47], autoimmune diseases [48–50] and cardiovascular diseases [51–53]. Despite the variety and complexity of modifications in the epigenetic landscape, many cancers exhibit a high degree of consensus across tissues, or within the tissue of origin [54–56]. The widespread nature of epigenetic changes across the genome can increase the sensitivity and specificity of this system in biomarker detection by utilizing multiple target loci in a single assay [14, 57]. In addition, the diagnostic sensitivity and specificity could be enhanced by combining the results of cirDNA methylation analysis with that of other cirDNA alternations, including copy number variations and point mutations. This integrated approach, while promising, will yield more information, requiring, in turn, a more advanced analytical methodology.

Despite several limitations, in recent years, cirDNA liquid biopsy has been performed for ctDNA methylation and mutation analyses [58–62]. In addition to the pre-analytical systems’ difficulty in isolating sufficient amounts of ctDNA, ctDNA methylation analysis in liquid biopsies involves several biological and technical drawbacks [63–65]. The main technical challenge is to isolate the very small quantities of cirDNA in liquid samples with biological variations [12, 66, 67]. Another significant difficulty in cirDNA-based biomarker selection is the careful detection of somatic target mutations/methylation aberrations from background signals [68, 69]. Recently, in the EU project SPIDIA4P, some main problems related to the pre-analytical variables for liquid biopsy analysis have been reported [70].

Even though the results available on clinical materials remain limited, several distinct methods have been explored to meet the growing demand for a clinically relevant technique for ctDNA/cfDNA methylation and mutation analysis [12, 71, 72]. The discovery of novel tissue-specific methylation markers for cirDNA detection requires complementary approaches. Next-generation sequencing (NGS) and polymerase chain reaction (PCR)-based analytical techniques are being continuously refined to accommodate methylation changes even with low frequencies [72].

Considered in the context of previous findings, our results revealed that these techniques are strongly influenced by the pre-analytical methods used to isolate cirDNA [23, 73, 74]. They also confirm our conviction that pre-analytical criteria and documentation are as critical as analytical requirements [24]. Indeed, the fact that each selected extraction system gives a different return of cirDNA [75, 76] confirms the ability of pre-analytical analysis to enhance the sensitivity of the detection phase in a routine clinical exercise.

To the best of our knowledge, there currently exists no guideline for pre-analytical parameters, and no standard operating procedure for the investigation of methylated cirDNA. In this article, we have conducted a comprehensive and systematic search for relevant pre-analytical variables and patient-related factors. These form the basis of the guidelines we propose for the analysis of methylated cirDNA.

## Material and methods

**Fig. 2:**
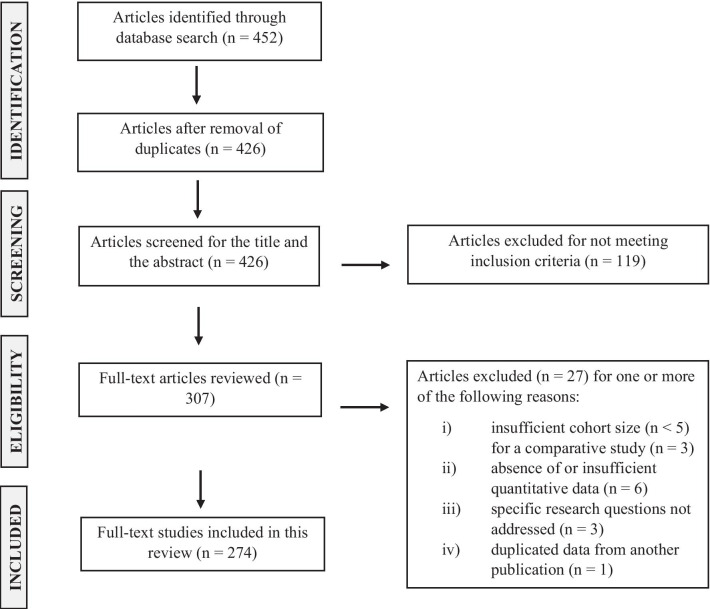
Flow diagram of literature search and inclusion/exclusion processes

We conducted a systematic review of the available literature search on pre-analytical and demographic parameters which could potentially influence the abundance of methylated cfDNA. We selected literatures published in the PubMed database from February 2000 to August 2021. As search terms, we used a combination of “circulating DNA”, “circulating cell-free DNA” and “methylation” with the following keywords: “pre-analytic”, “analytic”, “increased concentration”, “decreased concentration”, “blood collection”, “EDTA tube”, “cell-stabilizing blood collection tubes”, “plasma”, “serum”, “quantification”, “blood processing protocol”, “centrifugation methods”, “plasma quality control”, “extraction methods”, “isolation protocols”, “storage”, “blood stability”, “plasma stability”, “concentration stability”, “long-term storage”, “bisulfite conversion”, “bisulfite kit conversion”, “bisulfite conversion methods”, “bisulfite conversion efficiency”, “sequencing”, “detection methods”, “detection”, “analysis”, “stability of bisulfite converted cfDNA”, and “stability of bisulfite converted DNA”. Articles obtained from this search were further filtered for this review by careful analysis of the research purpose of each article and the study design quality. This selection process yielded 274 results. Two researchers (M.A. and M.A.K.) independently extracted the data. The literature searches and inclusion/exclusion processes are shown in Fig. 2.

## Results and discussion

The results of our systematic review have been summarized in 4 general and 9 specific challenges in order to optimize the pre-analytical phase of cirDNA methylation analysis.

A. **General challenges**
  1. **Blood collection and processing:** blood has broad inter-individual cirDNA variations; even an individual’s cirDNA varies during his/her life.
  2. **Blood volume:** a large volume of blood is often required to perform cirDNA isolation for methylation detection.
  3. **Blood storage:** blood is a complex biological material, sensitive to an assay period, in particular, due to enzymatic degradation or blood coagulation, it consequently requires strictly controlled storage conditions.
  4. **Choice of specimen type (plasma or serum):** blood has a heterogeneous content (containing different types of cells and free macromolecules), which impedes the isolation of cirDNA.
B. **Specific challenges**
  1. **Plasma isolation and preparation:** blood cells and cell organelles and debris must be removed from the blood.
  2. **Quality control of plasma:** DNA from blood cells or other sources may contaminate the target cirDNA.
  3. **Plasma storage:** the condition of plasma storage depends on the molecular structure of cirDNA.
  4. **cirDNA extraction methods:** the extraction method should be selected according to the therapeutic or industrial goals.
  5. **cirDNA integrity:** this is considered as the quality control of the extraction procedure.
  6. **cirDNA extract storage:** not recommended.
  7. **cirDNA bisulfite conversion:** optional.
  8. **Quality control of bisulfite converted cirDNA:** optional.
  9. **Storage of bisulfite converted cirDNA:** prolonged storage is not recommended.

A. **General challenges**
  1. **Blood collection and processing**
    A high level of inter-individual variation in plasma cirDNA concentration, known as “biological variation”, has been reported among patients [77]. As a result, the amount of cirDNA recovered from plasma can significantly differ from one methylation procedure to another [78, 79]. While some of these variabilities could be ascribed to biological differences between individuals, some relate to variations in the sensitivity of the different pre-analytical protocols applied in different laboratories. Moreover, some variabilities are caused by sources of DNA contamination, known as “technical variation”. The load of ctDNA also varies according to the source of blood collection. This could be explained by the clearance of cirDNA [80]. It is therefore important that not all blood samples be assumed equivalent, and that pre-analytical considerations be taken into account when prospective samples are being collected for ctDNA methylation analyses [81].
  2. **Blood volume**
    The volume of blood that may be collected in most clinical procedures is very limited; thus, there is typically only a relatively small level cirDNA available, which presents a challenge for the identification of methylation [82].
    Conventional techniques for cirDNA methylation detection from blood mostly require large volumes of blood samples (up to 12 ml), which must be used in the form of serum or preferably plasma for the extraction step [83]. It should be noted that the ideal volume of blood does not vary depending on the different extraction methods (e.g. column or bead-based kits). It is evident that the larger the volume of the blood sample, the higher the yield of cirDNA, although this yield will also depend on the capacity of the pre-analytical assay used for cirDNA isolation. Moreover, a fraction of ctDNA may be lost during bisulfite conversion. It is therefore important to collect a sufficient amount of the blood sample. Column-based isolation kits are not recommended for small volumes of plasma, since cirDNA could be trapped on the membrane and get lost [78].
  3. **Blood storage**
    Some researchers have reported that normal lavender-top tubes containing K2EDTA as an anticoagulant are appropriate for cirDNA isolation for methylation analyses [84, 85]. In general, the most widely employed anticoagulants include EDTA, heparin, and citrate-dextrose solution (ACD) [85]. Since heparin-containing samples exhibit dose-dependent interference with quantitative PCR (qPCR), various sample tubes can affect the data differently [86]. EDTA and ACD maintain cirDNA stability through inhibition of DNase activity and thus these anticoagulants, in particular EDTA, are most frequently used in genetic/epigenetic testing [87]. In general, no major difference in methylation status is likely to be introduced by the use of various anticoagulants, and no variation has been reported for methylation of DNA isolated from bloods collected into EDTA, heparin, and ACD tubes [88].
    However, it is safer to use EDTA tubes such as K2EDTA tubes to prevent the lysis of leukocytes. Moreover, the separation of plasma must be performed in the shortest practicable time. Several groups have documented the impact of delay between venipuncture and blood centrifugation on cirDNA levels. The general finding is that cirDNA levels increase with time prior to centrifugation of EDTA-stabilized blood [76, 89–91] and the accumulation of leukocyte DNA is a risk in EDTA tubes when blood is stored for more than 4 h. Recent studies have shown that EDTA tubes could also lead to variable results and therefore non-EDTA collection tubes have been produced to improve the cirDNA yield and to sustain quality over 7 days at ambient temperature [92–94]. Specialized blood collection tubes containing a preservative agent to prevent peripheral leukocyte lysis are available; these eliminate the need for rapid blood processing and enable storage of the whole blood at room temperature for several days until centrifugation [76, 95].
    While a variety of cirDNA collection tubes have been used for mutation analysis, it has been demonstrated that BD Vacutainer® K2EDTA, S-Monovette® K 3 E, and S-Monovette® CPDA tubes are appropriate for blood collection intended for analysis of plasma cirDNA methylation. These tubes allow the storage or shipment of blood at 15–25 °C for up to 48 h [96–98].
    Van Paemel et al. evaluated the efficiency of bisulfite-based cirDNA methylation profiling in various blood collection tubes, including BD Vacutainer K2EDTA tubes, and cell-stabilization tubes such as Streck Cell-Free DNA BCT, PAXgene Blood DNA, Roche Cell-Free DNA, and Biomatrica LBgard blood tubes, and they assessed the effect of the interval between blood drawing and plasma processing. Genome-wide bisulfite sequencing was conducted on the cirDNA isolated from plasma samples. They stated that the average methylation of CpG in EDTA tubes increased slightly (but not significantly) over time. However, the amount of CpGs covered with at least 15 reads was relatively stable. In contrast, the amount of CpGs with at least 15 reads diminished in Biomatrica, DNA Streck, and Roche tubes. They eventually concluded that the methylation pattern of cirDNA was stable and reproducible in all preservation tubes [99]. Bartak et al. reported that there was no significant variation between the K3EDTA and Streck tubes [100]. In general, the most cost-effective collection tubes are EDTA tubes; however, they must be handled within 4 h of blood collection (and the sooner the better), while cell-stabilized tubes (such as Streck tubes) may be used up to 8 days post blood draw at room temperature.
    We suggest that the plasma collection procedures for cirDNA methylation analysis should be optimized within each laboratory and that, depending on available infrastructures and facilities, researchers decide whether to process immediately or to use cell-stabilizing tubes to lower the risk of contamination from cellular fractions [101]. Furthermore, our advice is that plasma preparation should be carried out as early as possible: no longer than 4 h when using K2EDTA tubes, or within 48 h when using non-EDTA collection tubes. It is worth noting that storing blood samples in K2EDTA tubes at 4 °C will keep cirDNA levels sufficiently stable for 3 days for mutation detection [102–104] but not for methylation analysis.
    DNA yields and quality, which could influence the outcome of methylation assays, can be negatively affected by sample storage at higher temperatures [88], or by extended storage. (For example, storage for longer than 24 h at room temperature or even at 4 °C prior to DNA extraction may adversely affect the yield and quality of extracted DNA) [105, 106]. Although certain storage conditions have a significant impact on DNA extraction yield, little or no effect on the integrity and methylation of DNA has been reported [106]. A previous study showed that the storage of whole blood at 15 °C to − 80 °C for durations from 24 h to several months, or in liquid nitrogen, resulted in limited DNA methylation bias [105, 107]. Another study revealed that the storage of blood samples at room temperature for up to 24 h prior to isolation of plasma showed limited variation in CpG methylation (0.6%), and only a shift in methylation at particular sites [107]. Notably, a decrease in global DNA methylation occurred after 7 days of blood storage at room temperature [108]. There are limited numbers of studies, and further investigation is needed to confirm their results.
    Altogether, for cirDNA methylation studies, we recommend that blood be stored at 4 °C in K2EDTA tubes for up to 24 h [109–111]. In contrast, where nuclear cirDNA will be tested, plasma storage at − 80 °C is recommended [79, 112, 113].
  4. **Choice of specimen type (plasma or serum)**
    While serum may yield higher amounts of cirDNA than plasma, that yield is more variable, and quality of extracted DNA may be strongly affected by the additional amount of non-circulating (genomic) DNA generated by the lysis of monocytes and other blood cells due to the formation of blood clot. Consequently, plasma is less likely to be contaminated with the DNA of blood cells, and the interval between blood sampling and centrifugation should not be higher than 4 h to reduce this confounding factor [114]. As a result, cirDNA in serum is at least slightly polluted by genomic DNA derived by white blood cells, and thus, candidate cirDNA is diluted by high levels of non-specific genomic DNA. Thus, we conclude that while some types of cirDNA analysis may be carried out using serum, the use of plasma is certainly favored, owing to its low level of contamination with leukocyte DNA, maximizing sensitivity and data homogeneity [80].
    Previous studies have investigated cirDNA yield extracted from plasma and serum samples. Total cirDNA levels in serum were 1.63- to 11.09- fold higher than the plasma of healthy controls and cancer patients. The quality of cirDNA in plasma and serum has also been compared [115, 116], and cirDNA derived from serum has been shown to have significantly higher integrity than plasma, by using the ratio of 201 bp/105 bp amplicons (50% vs 33%, respectively). In the field of oncology, Thierry’s group have clearly shown that plasma is a better source of tumor-derived cirDNA. They indicated that the overall cirDNA concentration measured within a murine wild-type *KRAS* (non-tumor-derived cirDNA) was higher in serum samples than plasma samples using xenografted mice with human CRC cell line. When cirDNA concentrations were measured using a human wild-type *KRAS* (specific tumor-derived cirDNA), cirDNA concentrations were found to be higher in plasma samples. Their findings support the hypothesis that the rise in cirDNA in serum samples is due to blood cell DNA release [117].
    Although gene frequencies are lower in serum samples than in plasma [118], serum can nonetheless be a valuable source for extraction of ctDNA, because both plasma and serum exhibited comparable specificity (97% and 100%, respectively) and sensitivity (31% and 25%, respectively) [119]. El messaoudi et al. were the first to promote plasma over serum [114].
    Even though it has been known for a few years that plasma is preferable to serum, many research in the field still use serum samples, owing to clinical laboratories' proclivity for preparing sera traditionally and doing retrospective studies.
    However, we strongly recommend the use of plasma rather than serum in cirDNA methylation studies, since the background DNA could significantly influence the analytical results.
B. **Specific challenges**
  1. **Plasma isolation and preparation**
    The first step of plasma isolation refers to the traditional process, consisting of low-speed blood centrifugation to collect blood cells in the pellet, and a second, higher-speed centrifugation to remove cell organelles and debris [120]. While the use of several centrifugation steps does not significantly alter the yield of DNA [121], it does improve the homogeneity and purity of cirDNA for methylation detection. It has been suggested that the first centrifugation be performed at 4° C for 10 min at 800 to 1200 g, and the second step at 4 °C for 10 min at 14,000 to 16,000*g*. Other researchers have preferred to collect blood in EDTA tubes and to isolate plasma by double centrifugation at 2000*g* for 10 min [122], or by repeated centrifugation at 1350*g* for 12 min [123] followed by storage at − 80 °C until analysis [124]. Chiu et al. proposed the 1600 g 10 min/16000 g 10 min double centrifugation procedures [125].
    We advised that caution be exercised when collecting the first plasma supernatant to prevent contamination from the buffy coat. It should also be noted that, while filtration and fractionation on a Percoll gradient [125] are alternative approaches to the centrifugation method, their impact on the yields of cirDNA and cirDNA structures has as yet been insufficiently studied.
    Taken together, there is still no common protocol to prepare plasma for cirDNA extraction. However, as suggested by Chiu et al. the routine procedure is centrifugation at 1600*g* for 10 min at 4 °C, and subsequently at 16,000g for 10 min at 4° C [114, 126]. As the last step, aliquoting plasma in low volumes should be done immediately after separation. Low-volume aliquots guarantee the quality of the specimen during storage by reducing the number of freeze–thaw cycles. Typically, plasma aliquots in volumes of 300 µl–2 ml are used for cirDNA extraction and the analytical phase [24]. Recently, in a selective capture-based cirDNA isolation method developed by our group, cirDNA was isolated from 250 µl of plasma [127].
  2. **Quality control of plasma**
    The management of plasma consistency is of particular importance. Any traces of hemolysis can lead to an orange or red-colored plasma, which may indicate cell lysis. Hemolysis may lead to interference with PCR reaction. Icteric plasma can be distinguished by an extreme yellowish or greenish color, resulting from a high abundance of bilirubin. A strong interference with PCR reactions may occasionally occur with cirDNA taken from icteric plasma [78]. Moreover, it has been shown that plasma samples with opaqueness or turbidity contain a significantly lower concentration of cirDNA [77]. Samples can visually be compared against a typical hemolyzed specimen for the hemolysis rate [128]. We suggest that monitoring of plasma color should be carried out in plasma preparations after the first and the second centrifugation steps. Hemolytic, icteric, and opaque plasma samples should be declined, unless the blood sample cannot be substituted or no extraction method capable of isolating cirDNA from hemolytic blood is available [23, 129, 130].
    In our experience, hemolysis can be generated during blood sampling by the passage of blood through the needle, especially if it that is done too quickly, or due to the use of expired EDTA collection tubes. As a result, the lysed cells release their DNA in blood and increase the background DNA. The cell lysis could also inhibit the PCR reaction. To solve this problem in cases of cell lysis, we recommend using affinity capture beads for cirDNA isolation [97], although working on lysed-cell plasma is not recommended.
  3. **Plasma storage**
    cirDNA is subjected to a type of bloodstream homeostasis where the volume of identified cirDNA depends on the equilibrium between the degree of release and the extent of deterioration or clearance. Thus, plasma storage involves rapid cooling at 4° C, followed by preservation under frozen conditions to reduce nuclease activity. Several plasma cirDNA stability tests have been performed at either – 20 °C or – 80 °C [131]. Hebels et al. found that there is no systematic effect of anticoagulants (ACD, EDTA, or heparin) and storage temperature (- 80° C or liquid N2), on the yield of DNA or the quality of methylation profiles, if cirDNA samples extracted from plasma are stored at − 80° C within 8 h [107, 132].
    In general, to avoid cirDNA degradation, plasma must be stored at − 80° C; however long-term storage (> 3 years) may cause reduced cirDNA yield [23, 133]. Nevertheless, it is unclear whether or not samples of DNA stored for different periods below 4° C, particularly after years of storage, could retain stable methylation profiles [132].
  4. **cirDNA extraction methods**
    While cirDNA in the blood is most commonly used in liquid biopsy, cfDNA may also be extracted from different body fluids (including, but not limited to, saliva, urine, cerebral spinal fluid, or feces) [134]. The cirDNA extraction techniques for methylation detection vary from in-house procedures to the use of commercial kits. Of these, the QIAamp Circulating Nucleic Acid Kit (Qiagen, Hilden, Germany) is one of the most widely used [135–139]. Commercial kits (with or without automation) can be expected to obtain the necessary analytical homogeneity, yield, speed, and practicality for cirDNA extraction [140]. Bartak et al. concluded that, depending on the isolation approach, there will be variations in the quantities of cirDNA obtained for methylation detection [100]. In addition, a study conducted by Soriano-Tarraga et al. found some variations in global DNA methylation between different DNA extraction protocols (Autopure LIS, Qiagen; Puregen TM, Gentra Systems; Chemagic Magnetic Separation Module I, Chemagen); although these differences were not statistically significant [141].
    Today, most approaches are based on either magnetic beads or silica membranes. It should be noted that different tubes for extracting blood have varying criteria for extraction.
    In a recent study led by our group, following stringent standardization, the selective capture of cirDNA with magnetic beads showed a yield with a CV of 1.06%, compared with that of the silica membrane extraction method which was more variable (CV: 9.25%) [142]. Moreover, we have observed variations in the yield of extracted DNA between different commercial DNA extraction kits, depending on the size of the DNA fragment. Therefore, strict documentation of both the extraction method and the quantification method is needed. It is worth noting that alternative approaches for cirDNA extraction are currently under development [143], and that existing reports on the direct quantification of total cirDNA concentration do not include a full blood extraction procedure [77, 144–146].
    To determine the effect of the DNA concentration on the DNA recovery from plasma samples, serial concentrations of the spiked DNAs (sheared genomic DNA with sonicator) in plasma can be prepared, extracted, and analyzed by qPCR [147]. Adding a spike plasmid material to plasma as a control before extraction can be used to measure the extraction efficiency and the performance for different fragment sizes [148]. Basically, DNA shearing is done using an ultrasonicator [149] or enzymatic digestion [150]. However, cfDNA from cancer patients differs from sheared DNA fragments. It has been shown that cfDNA exhibits certain characteristics of nucleosomal DNA and positioning, while randomly sheared fragments do not possess such properties [151].
    As a result of the various fragmentation patterns, ultrasonication fragments may not be the desired method.
  5. **Quantification methods and cirDNA integrity**
    qPCR is currently the most widely used method for cirDNA quantification, while spectrophotometry and fluorometry (e.g. PicoGreen and Qubit) are also used as cost-effective, rapid, and simple methods [76, 152]. By using small volumes of samples, NanoDrop allows the preservation of DNA samples. However, the absorbance calculation is limited to a minimum of 2 ng/µl double-stranded DNA concentration, which is typically not affordable with minute concentrations of cirDNA extracts. Fluorimetric quantification is the preferred option for analyzing samples with such a relatively low cirDNA concentration [153, 154]. qPCR-based methods, however, provide the highest levels of sensitivity and specificity for DNA concentration measurement [155, 156]. Ramachandran et al. compared fluorometry and UV spectrophotometry with qPCR and reported that while cirDNA levels quantified by qPCR correlated with those measured with PicoGreen assay, they did not correlate with the ones determined by UV spectrophotometry (*r* = 0.23) [157]. Van Ginkel et al. indicated a positive correlation of dPCR with Qubit fluorometry (*r* = 0.98; *P* < 0.0001) but not with UV spectrophotometry (conducted using a Nanodrop) [158].
    Droplet digital PCR (ddPCR) is a technology that overcomes previous technological challenges and allows for the identification and absolute quantification of nucleic acid targets in a variety of clinical specimens, creating new possibilities for developing DNA methylation-based disease biomarkers [159, 160]. Absolute concentrations of DNA may be quantified using ddPCR technologies, utilizing automated measures rather than calibrating an analog signal with a linear curve, as is done in traditional or optimized qPCR approaches [161]. When used in strict optimized conditions, qPCR is as sensitive and specific as ddPCR [63]. The quality of the primer design is crucial in determining whether or not qPCR can detect a reduced number of DNA fragments. Two main qPCR drawbacks should be considered when quantifying cirDNA. First, qPCR cannot measure cirDNA fragments smaller than the amplicon size produced by the qPCR priming method. For example, due to the characteristics of an efficient priming system, amplicons smaller than 45 bp cannot be detected. Thus, given the large variability of cirDNA fragment sizes, quantification strictly depends on the amplicon size [24, 162, 163]. Second, because qPCR methods are designed for a specific sequence, they may discern and separately measure only specific fragments of cirDNA extracted from nuclear or mitochondrial sources. In contrast, spectrometry and fluorimetry enable quantification of the total amount of cirDNA derived from both sources. Notably, the use of fluorophores to quantify very short cirDNAs (< 120 bp) is not streamlined, owing to the lack of linearity in analytics. However, this is the concept of Qubit, which is CE-IVD (CE in vitro diagnostic) marked within the workflow of the Clarigo test, a highly accurate non-invasive prenatal test (NIPT) based on Agilent’s Multiplex PCR technology [79, 164].
    The quality of cirDNA can be tested by determining the extent of fragmentation. We presented several methods of evaluating and validating cirDNA isolation [163]. A handful of studies have suggested that the DNA integrity index could be determined by the ratio of qPCR products using primer sets specifically for 80 bp and 250 bp amplicons [63, 165]. This fragmentation index, ranging from 0.3 to 0.8, demonstrates the level of homogeneity of cirDNA samples. Highly fragmented or contaminated samples can be declined [63, 136].
  6. **cirDNA extract storage**
    DNA degradation typically increases upon dilution, repeated freeze–thaw cycles, and longer storage [114, 133, 166, 167]. While contamination with nucleases must be prevented when treating DNA, chemical deterioration poses the main threat to DNA integrity [165, 168, 169]. Furthermore, the small size of fragments may alter the effectiveness of quantification and detection or may lead to negative results, depending on the analytical method used. Precautions should therefore be taken to inhibit any shortening of DNA that might occur during storage [79, 87].
    While it has been suggested that, in addition to storing cirDNA in solution at very low temperatures, cirDNA might also be stored in a dried state. However insufficient data on such a technique currently exists. If the analytical assessment has to be delayed, it is recommended that cirDNA extracts be stored at – 20 °C or − 80 °C, with no more than three freeze–thaw cycles.
    In our experience, storing cirDNA at − 20 °C for longer than 1 year could have an adverse effect on DNA methylation, and we, therefore, do not recommend this.
  7. **cirDNA bisulfite conversion**
    The need for a bisulfite conversion (BSC) step prior to biomarker evaluation is a particular concern when using cirDNA-based methylation biomarkers. This step allows for discrimination between methylated and unmethylated cytosines. Notably, a significant amount of DNA is often lost during bisulfite conversion due to chemical degradation of DNA and suboptimal post-conversion purification processes [170–175]. It has been reported that the lower input of amplified bisulfite-converted DNA (10 vs. 50 ng) results in a reduction of the 450 K array (Illumina) methylation signal [176]. The loss is apparently greater for low molecular weight (LMW) than high molecular weight (HMW) cirDNA [170, 177, 178]. Only a small number of studies, however, have addressed the difficulties of obtaining reasonable levels of cirDNA recovery and performance upon bisulfite conversion of cirDNA [179–181].
    After bisulfite treatment, chain breakage of cirDNA produces fragments smaller than its original average size of 180 bp [182], which limits the subsequent detection step. Fragmentation significantly affects cirDNA recovery after treatment with bisulfite. When the starting quantity of DNA is low, most of DNA treated with bisulfite is lost during purification by standard methods [170, 182–184], particularly when using a column-based approach [142]. At high incubation temperatures, one major drawback of bisulfite treatment is DNA degradation. Thus, the original technique has been modified over the years to simplify the conversion procedure [182, 185–188]. Several companies offer bisulfite conversion kits, some of which rely on high-capacity magnetic beads; however, they still cost over 200 € per 96 samples [189]. Several studies have introduced a rapid deamination step in bisulfite conversion, reducing the incubation time from 12–16 h to 40 min using a highly concentrated solution of bisulfite at higher temperatures [180, 185, 190]. This optimized approach allows more homogenous processing of cytosine in a short time [177, 191, 192]. Generally, if the size of DNA fragments is important for the subsequent procedure, temperature and incubation time should be adjusted [182].
    Several recent studies have shown that the bisulfite conversion method determines the purity of cirDNA, and that there is a trade-off between achieving a high yield and reducing contamination with inhibitors [23, 184, 193, 194]. Currently, most bisulfite conversion methods rely either on magnetic beads or a solid substrate, such as silica membranes. While solid substrate approaches have been introduced to simplify the process, magnetic beads have shown excellent potential for standardizing current purification procedures [195–197]. In addition, the magnetic bead-based method is efficient in terms of the overall yield and the purity of isolated bisulfite-converted ctDNA with only a very small range of variations [198–202]. We have recently shown that both cirDNA isolation and the bisulfite process could be conducted in a single tube using a bead-based approach (unpublished data).
    It should be noted that there are also several analytical methylation assays, independent of bisulfite treatment, including MeDIP-Seq [203], MBD-Seq [204], aka hMe-Seal [205], and Methylsorb [206].
  8. **Quality control of bisulfite converted cirDNA**
    Most analytical approaches for DNA methylation have focused on bisulfite conversion of genomic DNA. After the bisulfite treatment, the success of all methods depends on the DNA quality. The evaluation of the quality of DNA treated with bisulfite can be done using HPLC or gel-based assays. These assays require large quantities of DNA and consume most of the product of a single bisulfite conversion reaction [207, 208]. Gel-based approaches require approximately 2 μg of DNA. When analyzing converted DNA by agarose gel electrophoresis, it is recommended that a 2% gel with a 100 bp DNA marker be used. Typically, loading up to 100 ng of a sample may be needed to visualize the converted DNA. Many researchers are concerned about not finding any band on agarose gels, since the majority of the concerted DNA is single-stranded. To resolve this problem, the gel must be cooled in an ice bath for a few minutes prior to the run. This will drive base pairing between the single-stranded DNA molecules; the recovered material can consequently be marked and illuminated by the DNA intercalators. The recovered DNA typically smears on the gel from 1,500 bp down to 100 bp [209]. Ultraviolet (UV) or fluorescence-based approaches allow for quantification of the gel but do not provide information on DNA degradation [172]. Ehrich et al. have proposed a new method to assess the quality of the bisulfite-converted DNA. The assay consists of four amplicons with increasing lengths located in the IGF2/H19 region and combines analysis of the effectiveness of the amplification with an estimation of the methylation level variation to provide a practical estimation of the DNA quality [207]. Alternatively, using a reference gene with its specific primers and probes could also indicate the quality of extraction and the DNA integrity after bisulfite conversion in a real-time PCR assay [14].
    When using spectroscopy for quantification, a value of 40 μg/ml for Ab260 nm equal to 1.0 should be used, since the converted DNA behaves more like RNA. Some researchers may find their recovery very low. There are two reasons for this: 1) The DNA recovery is extremely low; this is because it is possible to lose DNA samples during the bisulfite treatment process, particularly if degraded DNA is used. 2) The initial quantification is inflated with RNA contamination. RNA is then removed during the bisulfite processing and clean-up phases, and the level of quantification in the subsequent step therefore appears to be lower [209]. In either case, the recovered material is generally still sufficient, especially if a downstream PCR step is planned. Typically, starting with RNA-free intact DNA gives the best results [207]. In addition, as a quality control step, the bisulfite-converted DNA may be used to conduct PCR with standard non-bisulfite-specific primers to amplify any unconverted product [210].
  9. **Storage of bisulfite converted cirDNA**
    Highly stability in the bisulfite-treated DNA is beneficial if studies are to be performed over a prolonged period. That stability varies according to which brand of bisulfite conversion kit is used (Table 1).

**Table 1:** The stability of bisulfite converted DNA prepared by different commercially available kits

Kit Name	Input volume, µl	Elution volume, µl	Input DNA	Optimal input DNA	Size	Recovery (%)	Efficiency (%)	Source	DNA protection	Protocol time (min)	Storage time	96-Well format	Automation
MethylEasy Xceed	20	12–100	0.05 ng–5 µg	0.05 ng–5 µg	–	> 99.9	> 99	Cells, tissue	Yes (Salmon sperm)	90	> 3 months	Yes (beads)	–
CpGenome™ Turbo Bisulfite Modification Kit	10	25 + 25	0.5 ng–1 µg	1 ng–1 µg	–	–	> 99.9	Cells, tissue	No	100	> 2 months	No	–
EpiTect Plus Bisulfite Conversion	20–40	20 + 20	1 ng–2 µg	1–2 µg	–	–	> 99	All kinds	Yes (carrier RNA)	370	18 months	Yes	Yes
Bisulflash™ DNA Modification Kit	1–5	10–20	0.2 ng–1 µg	50–200 ng	> 100 bp, optimized for 250 bp	> 90	> 99.9	–	No	60	6 months	Yes	Yes (beads)
EpiJet Bisulfite Conversion Kit	20	6–20	0.05 ng–2 µg	200–500 ng	Optimized for > 50 bp	–	> 99	–	No	220	1 year	No	Yes
Imprint DNA Modification Kit	10	8–20	0.1 ng–1 µg	50–200 ng	–	–	> 99	–	Yes (BSAsolution)	155	2 months	No	No
EpiMark Bisulfite Conversion Kit	10	20 + 20	50 ng–2 µg	50 ng–2 µg	Optimized for > 150 bp	–	–	–	No	245	6 months	No	No
EZ DNA Methylation Direct Kit	20	10	50 pg—2 µg	200–500 ng	> 17 bp	> 80	> 99.9	All kinds	No	260	6 months	Yes	Yes (beads)
Premium Bisulfite	20	10	0.1 ng–2 µg	200–500 ng	Optimized for bp > 500	> 80	> 99	–	No	115	6 months	No	Yes (column)
Bisulflash™ DNA Bisulfite Conversion Easy Kit	2–15	10–20	100 ng–1 µg	100 ng	> 100 bp, optimized for 250 bp	> 75	> 99.9	Cells, tissue	No	80	6 months	Yes	No
innuCONVERT Bisulfite Body Fluids Kit	50	20–100	0.5 ng–10 µg	–	Optimized for > 100 bp	–	–	cirDNA in body fluids	Yes (carrier RNA)	110	2 months	No	–
Custom made Protocol	35	35	–	–	> 17 bp	–	–	–	No	50	6 months	No	Yes
    In general, based on the commercial bisulfite conversion kits, converted and purified DNA can be stored at − 20 °C for at least 6 months with no significant loss of quality [208, 211–214].
    The preanalytical guidelines have been designed to fit different empirical endpoints, according to their specifications shown in Fig. 3.

**Fig. 3:**
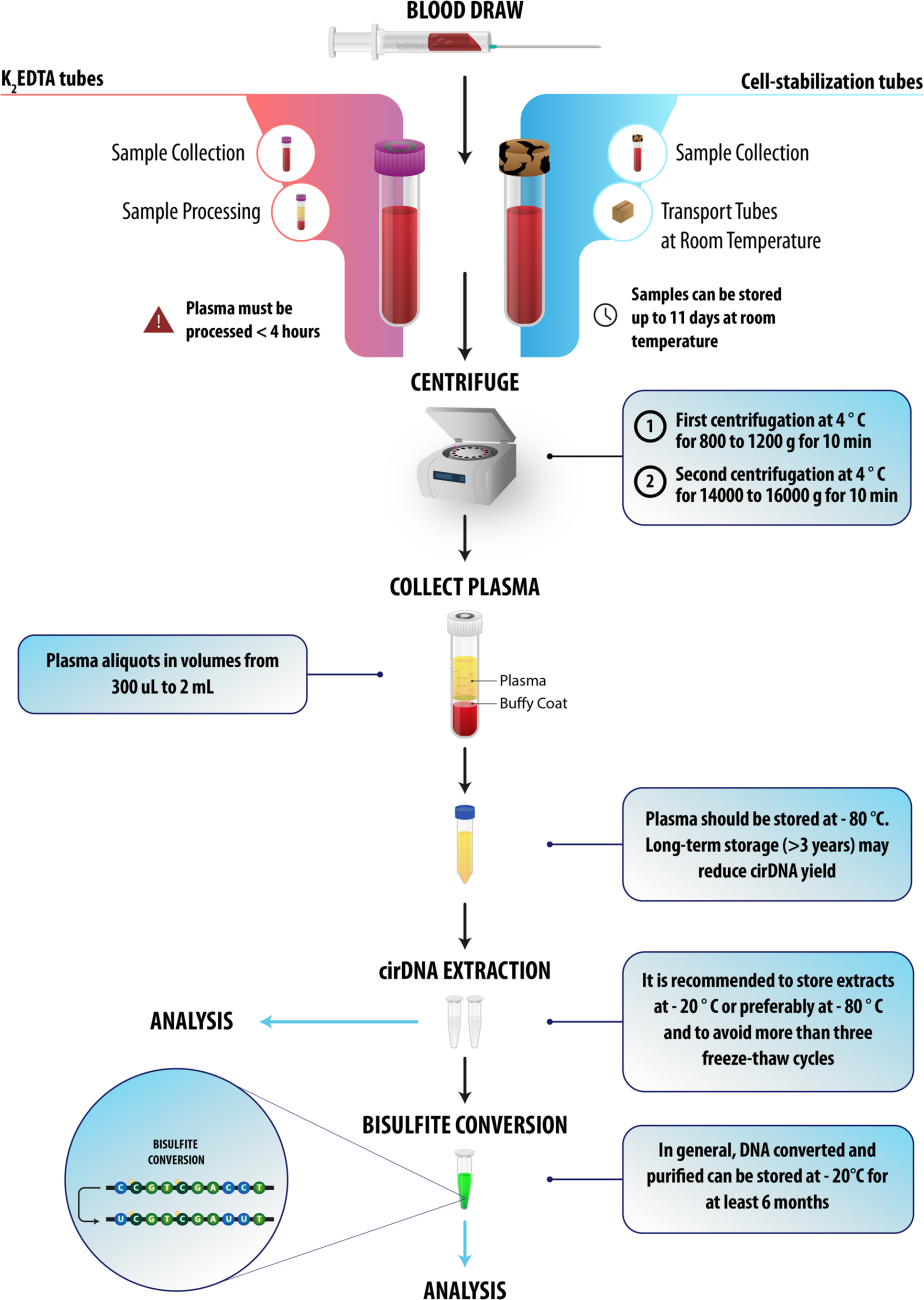
A pre-analytical guideline for cirDNA methylation analysis

### Analytic phase

DNA pre-amplification methods are commonly used to reach minimum input criteria for downstream molecular analysis in mutation detection. Targeted pre-amplification of a stretch of cirDNA containing a mutation has previously been shown to generate appropriate template amounts for ddPCR [215]. However, it is applicable for methylated cirDNA only after bisulfite conversion.

Whole-genome amplification (WGA) methods have been optimized for naturally short and fractured cirDNA models, particularly when cirDNA is present in only finite or trace quantities [216]. Different WGA kits use a sample input of 5–10 ng. Multiple displacement amplification (MDA) is a recently described method of WGA that has proven efficient in the amplification of small amounts of DNA, including DNA from single cells (the amount of 6.6 pg) [217]. Since MDA could analyze long and intact genomic DNA from single-cell genomes, it is not suggested for cirDNA [218].

### cirDNA methylation detection methods

**Fig. 4:**
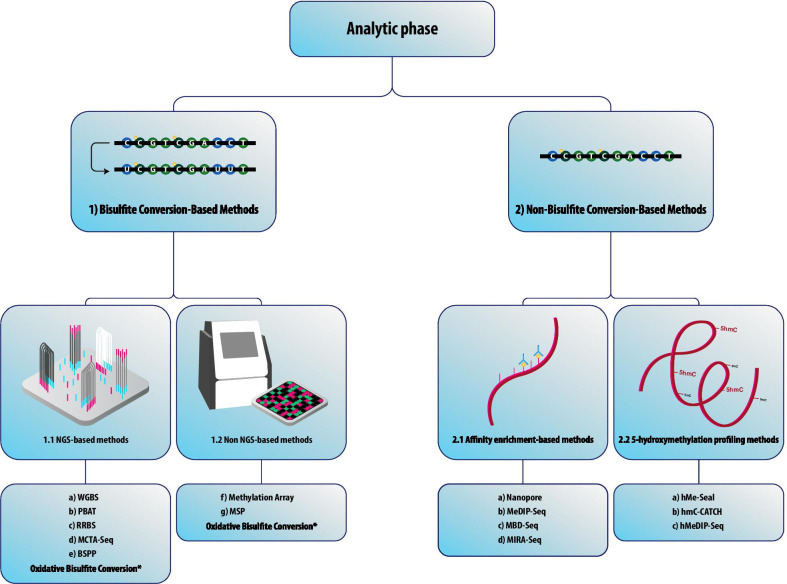
cirDNA Methylation Detection Methods. Methods for the detection of cirDNA methylation can be divided into two groups: (**1**) Methods based on bisulfite conversion. These techniques are presented as the gold standard for DNA methylation studies. Bisulfite conversion typically causes significant deterioration of DNA, resulting in the loss of some essential information, especially when the input cirDNA is low [183]. Bisulfite conversion methods include: (**a**) Whole-Genome Bisulfite Sequencing (WGBS), initially established to map the human DNA methylomes [223]. WGBS is currently the most comprehensive DNA methylation profiling technology [224] with the ability to identify cytosines’ methylation state, including regions with low CpG density as well as non-CpG sites (CpA, CpT, and CpC). It is an expensive technique, especially when producing high-depth data, which requires only 30 ng of human input DNA. Other approaches, such as single-cell bisulfite sequencing (scBS-Seq) [225] and single-cell whole-genome bisulfite sequencing (scWGBS), have been established to address the rising demand for low-input DNA analysis [226]. (**b**) Post-bisulfite adaptor tagging (PBAT) is a very effective method for creating libraries for WGBS with low input DNA. PBAT adds adapters to bisulfite-converted genomic DNA to bypass the DNA degradation inherent in standard WGBS protocols, triggered by bisulfite. PBAT is a PCR-free approach that adds adaptors after bisulfite treatment to avoid bisulfite-induced DNA library degradation, thus it can create a library starting from 125 pg of DNA [227, 228]. (**c**) Reduced-Representation Bisulfite Sequencing (RRBS) was employed to make DNA methylome analysis cost-effective by combining MspI digestion and bisulfite conversion for the study of regions enriched in CpGs, which requires extremely low input DNA (10–300 ng) [229, 230]. (**d**) Methylated CpG Tandems Amplification and Sequencing (MCTA-Seq) is a highly sensitive method for the identification of hypermethylated CpG islands [231] that uses a primer harboring a semi-random sequence, a special molecular identifier (UMI) sequence, and an anchor sequence, to amplify the DNA transformed with bisulfite at the 3'-end.. This highly sensitive technique will work with as little as 7.5 pg of genomic DNA, which is equal to 2.5 copies of the haploid genome [232]. (**e**) Bisulfite padlock probes (BSPP), where bisulfite-converted DNA is isolated using specific probes which contain two short capture sequences connected via a standard linker sequence [233, 234]. This method can be performed on low DNA input as low as 10–15 ng [235]. A unique feature of BSPP is its ability to be incorporated into a capture reaction across hundreds of samples resulting in more than 97% specificity. (**f**) Methylation array: Illumina’s MethylationEPIC ‘850 K’ Bead Chip is an excellent tool for quantitative methylation analysis at a single CpG site level. It enables the interrogation of over 850,000 methylation sites, selected by experts [236]. One drawback of array-based approaches, however, is the low genome-wide coverage of entire methylation regions, leading to the lack of some genome methylation contents [237]. (**g**) Methylation-specific PCR (MSP) where two different methylation-specific primer sets against target DNA are used to amplify methylated DNA converted with bisulfite and untreated DNA. In an unmethylated state, the unmethylated primer is unique to bisulfite converted DNA [238]. Several quantitative MSP (qMSP) methods have been established using real-time PCR [239–241]. For methylation detection, the methylation-sensitive high-resolution melting analysis (MS-HRM) technique has also been created [242]. However, our group has found that optimizing this method can be laborious and, in some cases, may have unreliable. (**2**) Methods based on non-bisulfite conversion. In order to overcome the limitations of bisulfite conversion, several non-bisulfite conversion techniques have been developed. The restriction enzyme-based method is a conventional approach for methylation analysis, which commonly uses two types of methylation restriction enzymes (MREs): methylation-sensitive enzymes, which cleave only unmethylated DNA; and methylation-insensitive enzymes, which cleave DNA regardless of the methylation status at the recognition sites [172]. Non-bisulfite conversion methods are categorized as (**2.1**) Affinity enrichment-based methods, where anti-methylcytosine antibodies or methyl-CpG binding proteins are used to pull down the methylated genomic fragments, while stringent washing eliminates unmethylated fractions. These enrichment-based approaches have not only demonstrated comparable sensitivity to and marginally greater precision than WGBS methods but have also demonstrated additional benefits [243]. They include: (**a**) Nanopore sequencing technology makes significant advances in the detection of DNA methylation with an accuracy of 92–98%. It works based on immersing a high-resistance film with protein nanopores in an aqueous ion solution[244–246]. (**b**) Methylated DNA Immunoprecipitation Sequencing (MeDIP-Seq) was initially established as a method for the immunoprecipitation of methylated DNA [203]. Due to inadequate methylation enrichment, a minimum input of 50 ng DNA is recommended [247] and in practice, the DNA amount in plasma limits the application of this method. Cell-free methylated DNA immunoprecipitation and high-throughput sequencing (cfMeDIP-Seq) has been established to employ MeDIP-Seq for low-input cirDNA [67]. (**c**) Methyl-CpG Binding Domain Protein Capture Sequencing (MBD-Seq). The methyl-CpG binding domain in methyl-CpG binding proteins may be used instead of immunoprecipitation to pulldown DNA methylated regions using magnetic beads [204]. MBD-based enrichment has been shown to outperform MeDIP in regions with a high CpG density and to classify the largest proportion of CGIs [248]. (**d**) Methylated CpG Island Recovery Assay (MIRA-Seq) that works based on the MBD2b/MDB3L1 complex high affinity for double-stranded methylated DNA, enabling the isolation of methylated DNA without the use of bisulfite conversion or antibodies. MIRA can detect methylated CpG nucleotides with low methylation density and can be used in combination with microarrays or NGS [249]. (**2.2**) 5-Hydroxymethylation profiling methods. Emerging data suggest that 5hmC not only serves as a generally stable epigenetic marker [250], but also interacts with tumorigenesis and tumor development [251]. They include: (**a**) 5hmC-Seal (aka hMe-Seal) where azide-modified glucose is produced by β-glucosyltransferase and then biotinylated through click chemistry. Since 5hmC-Seal can work with ultra-low amounts of input DNA (about 5 ng), this technology is very useful for liquid biopsy [252, 253]. (**b**) hmC-CATCH is a bisulfite-free method for genome-wide identification of 5hmC requiring only nanoscale input genomic DNA samples [254]. This approach is based on the 5-formylcytosine (5fC) blocking method, 5hmC to 5fC selective oxidation, newly created 5fC chemical labeling, and C to T transformation during PCR amplification [254]. (**c**) Hydroxymethylated DNA Immunoprecipitation Sequencing (hMeDIP-Seq) method is an updated version of MeDIP that enables the unique enrichment of DNA fragments harboring 5hmC [255]. hMeDIP requires immunoprecipitation using anti-5hmC antibodies accompanied by downstream approaches including NGS, microarray, or PCR. (**d**) Oxidative Bisulfite Conversion. Cytosines in 5fC and 5-carboxylcytosine (5caC) are not preserved after deamination by sodium bisulfite; this led to the development of oxidative bisulfite sequencing (OxBS-Seq) [256] and TET-assisted bisulfite sequencing (TAB-Seq) [257]

DNA pre-amplification methods are commonly used to reach minimum input criteria for downstream molecular analysis in mutation detection. In general, cirDNA methylation detection methods can be divided into bisulfite conversion- and non-bisulfite conversion-based techniques. Given that the focus of this review was the pre-analytic phase, we only provide a brief overview of the recent analytic methods for the detection of cirDNA methylation in Fig. 4.

## Conclusions

We have systematically reviewed current literatures on cirDNA to explore the influence of pre-analytical factors in the methylation analysis of cirDNA on the quality and/or yield of cirDNA.

The guidelines of pre-analytic stages for methylation and mutation detection are different since methylation in contrast to mutation is not stable as it could be lost during PCR reactions. Methylation detection of ctDNA fragments is challenging because of the low abundance of methylated nucleic acids compared with background DNA [198, 219]. Methylation detection of cirDNA usually requires several laborious steps, as well as transfers between multiple tubes, thus resulting in the loss of samples, increased contamination, high operator error rates, and data variation [220]. Its analysis remains a fragmented process, since DNA extraction, bisulfite treatment of DNA, and methylation detection are carried out separately [196].

It had recently been documented that, for pre-analytical variables, the choice of blood collection tubes [78], cirDNA extraction [221], and bisulfite conversion methods could affect the detection of cirDNA methylation [222]. The fact that each selected system has a different return of cirDNA [75] confirms the importance of pre-analytical analysis in enhancing the sensitivity of the detection phase for a routine clinical exercise [63].

In this review, we have summarized the methodologies that we recommend as best practices in cirDNA processing for methylation analysis. It helps research groups to identify variability among different methods for cirDNA methylation analysis, and further promotes the application of cirDNA in multi-center clinical trials as a step towards clinical adoption.

To sum up, the choice of pre-analytical variables can affect the identification of cirDNA methylation. Indeed, different techniques give a different return of cirDNA, which confirms the importance of pre-analytical procedures in clinical diagnostics.

## Data Availability

Not applicable.

## Abbreviations

cirDNA: Circulating cell-free DNA
ctDNA: Circulating tumor DNA
cfDNA: Cell-free DNA
NGS: Next generation sequencing
PCR: Polymerase chain reaction
qPCR: Quantitative PCR
LMW: Low molecular weight
HMW: High molecular weight
MREs: Methylation restriction enzymes
WGBS: Whole-genome bisulfite sequencing
scBS-Seq: Single-cell bisulfite sequencing
scWGBS: Single-cell whole-genome bisulfite sequencing
RRBS: Reduced-representation bisulfite sequencing
CGIs: CpG islands
PBAT: Post-bisulfite adaptor tagging
MCTA-Seq: Methylated CpG tandems amplification and sequencing
BSPP: Bisulfite padlock probes
MSP: Methylation-specific PCR
qMSP: Quantitative MSP
MS-HRM: Methylation-sensitive high-resolution melting
MeDIP-Seq: Methylated DNA immunoprecipitation sequencing
MBD-Seq: Methyl-CpG binding domain protein capture sequencing
MIRA-Seq: Methylated CpG island recovery assay
CRC: Colorectal cancer
NSCLC: Non-small-cell lung cancer
HCC: Hepatocellular carcinoma
5fC: 5-Formylcytosine
hMeDIP-Seq: Hydroxymethylated DNA immunoprecipitation sequencing
5caC: 5-Carboxylcytosine
OxBS-Seq: Oxidative bisulfite sequencing
TAB-Seq: TET-assisted bisulfite sequencing
HPLC: High‐performance liquid chromatography
